# Predation risk and space use of a declining Dall sheep (*Ovis dalli dalli*) population

**DOI:** 10.1371/journal.pone.0215519

**Published:** 2019-04-15

**Authors:** Catherine Lambert Koizumi, Andrew E. Derocher

**Affiliations:** 1 Department of Biological Sciences, University of Alberta, Edmonton, Alberta, Canada; 2 Gwich’in Renewable Resources Board, Inuvik, Northwest Territories, Canada; Université de Sherbrooke, CANADA

## Abstract

The abundance of ungulate populations may fluctuate in response to several limiting factors, including climate, diseases, and predation. In the northern Richardson Mountains, Canada, Dall sheep (*Ovis dalli dalli*) have undergone a major decline in the past decades and predation by grizzly bears (*Ursus arctos*) and wolves (*Canis lupus*) was suspected as a leading cause. To better understand the relationship between these three species located in this rugged and remote ecosystem, we relied on a combination of indirect methods. We investigated the apparent role of predation on the Dall sheep population using spatial ecology and stable isotopes. We examined seasonal variation in predation risk, focusing on how it may affect Dall sheep habitat use and sexual segregation, and we evaluated the proportion of Dall sheep in the diet of both predators using stable isotopes. The movements of the three species were monitored by satellite telemetry. Dall sheep habitat use patterns were analyzed using topographical features, greenness index, land cover, and apparent predation risk. The diets of grizzly bears and wolves were examined using a Bayesian mixing model for carbon and nitrogen stable isotopes. We found that Dall sheep habitat use varied seasonally, with different patterns for ewes and rams. Exposure to grizzly bear risk was higher for rams during summer, while ewes were further exposed to wolf apparent predation risk during winter. The importance of safe habitats for ewes was reflected in space use patterns. Stable isotopes analyses suggested that the diet of grizzly bears was largely from animal sources, with mountain mammals comprising about one quarter. Wolves mostly fed on both aquatic browsers and mountain mammals. Diet variation between individual predators suggested that some individuals specialized on mountain mammals, likely including Dall sheep. We conclude that grizzly bear and wolf apparent predation risk are important in driving Dall sheep habitat use and play a role in sexual segregation. Overall, this study presents an innovative combination of indirect methods that could be applied elsewhere to better understand predator-prey dynamics in remote ecosystems.

## Introduction

Determining the effects of predators on a prey population is an enduring ecological challenge, especially for wide-ranging species in secluded areas. Quantifying predation events in remote areas may be prohibitively expensive and logistically challenging. However, the investigation of indirect effects of predation is more tractable and can reveal various insights into predator-prey interactions [1] because predator avoidance or predation risk may have a significant influence on habitat use patterns [2,3]. In a landscape of fear, predation risk may also lead to sexual segregation, resulting in males frequenting habitats of higher foraging quality to increase their reproductive success while females, focused on protecting offspring from predators, stay in safer areas, even if those offer lower nutritive value [4,5,6,7].

In this study, we examined the risk posed by grizzly bears (*Ursus arctos*) and wolves (*Canis lupus*) on a remote Dall sheep (*Ovis dalli dalli*) population in the northern Richardson Mountains, Northwest Territories and Yukon Territory, Canada. This population is located at the northeastern limit of the species range and had been declining for fifteen years. Aerial surveys revealed that the population increased in the 1980s [8] to 1400, 1700 in the 1990s [9,10], then rapidly declined to about 700 animals in the early 2000s [11,12,13], and to only 497 individuals in 2014 [13]. Factors that may contribute to this include human harvest, climate change, competition (intraspecific and interspecific), predation and, to a lesser degree, parasites and diseases [14]. Infectious pneumonia that caused die-offs of bighorn sheep (*O*. *canadensis*) at lower latitudes [15,16] has not been reported in Dall sheep. Grizzly bears and wolves are the two main predators of the Richardson Mountains population [8,17] although their reliance and potential effect on Dall sheep population dynamics remains uncertain. In this paper, our objectives were to determine how apparent predation risk, measured as spatial overlap using satellite telemetry, varied seasonally, and how it may be associated with Dall sheep individual habitat use and sexual segregation. We hypothesized that predation risk of grizzly bears and wolves influenced Dall sheep habitat use and sexual segregation. As such, we expected Dall sheep rams to use habitats of higher nutritive quality and be exposed to greater predation risk levels than ewes most of the year. We expected ewes to use habitats of lower apparent predation risk than rams, particularly perinatal, despite potentially lower forage quality. We tested those predictions by comparing Dall sheep rams and ewes seasonal habitat use patterns under predation risk. We also examined the presence of Dall sheep in the diet of grizzly bears and wolves using stable isotopes analyses. Naturally occurring stable isotope ratios of carbon and nitrogen in consumer tissues can help estimate the proportion of assimilated food sources and reconstitute food webs [18,19]. For instance, *δ*^13^C and *δ*^15^N stable isotopes have been used to distinguish diet variability, identify foraging profiles, and quantify the importance of several food sources into the diet of wolves [20,21,22] and grizzly bears [23,24,25]. By integrating spatial ecology with isotopic diet analysis, we provide insights into predator-prey relationships in this remote ecosystem.

## Study area

**Fig 1:**
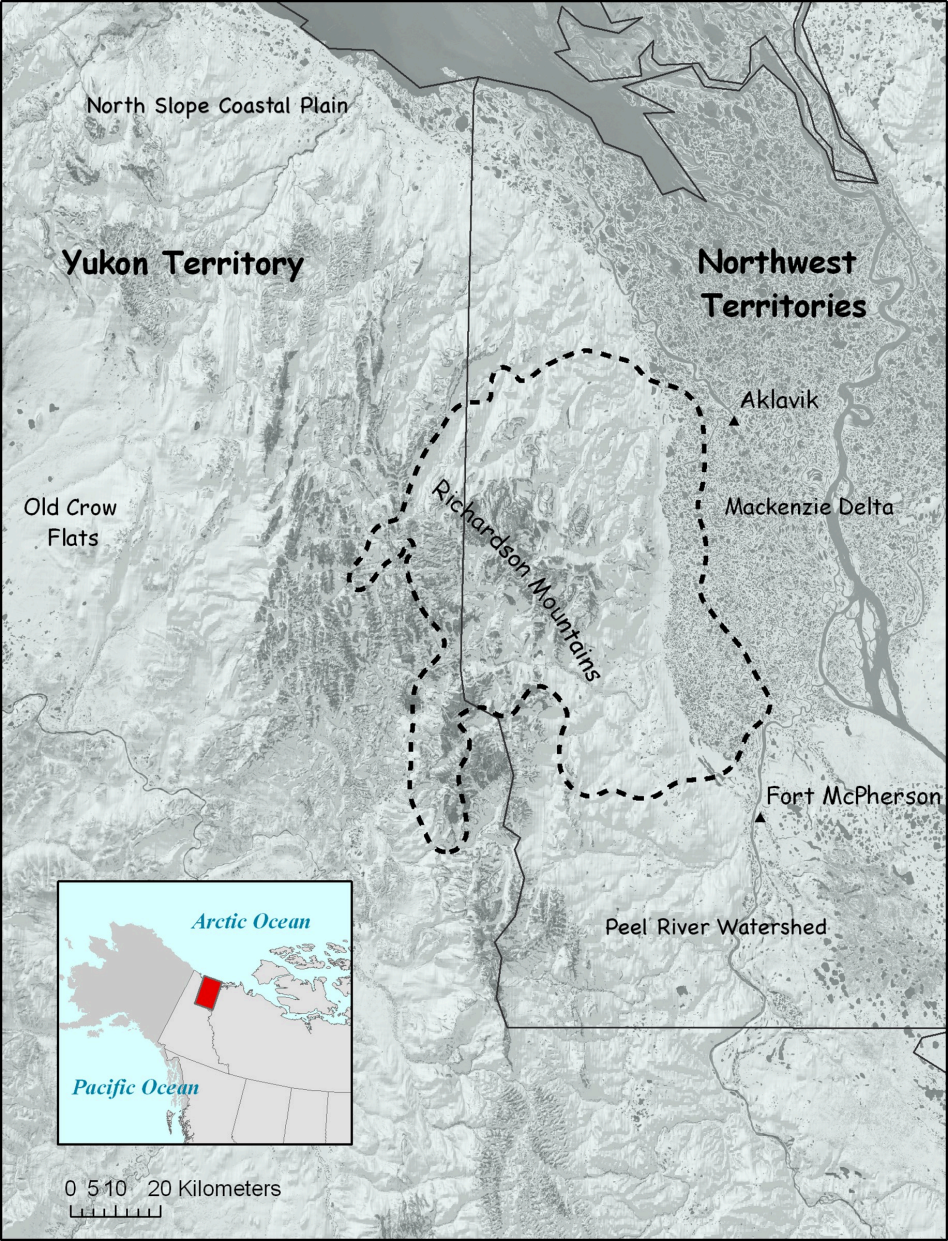
Study area in the northern Richardson Mountains, Northwest Territories and Yukon Territory, Canada.

The study area was located in the northern Richardson Mountains in the Canadian Arctic in the Northwest Territories and the Yukon Territory (Fig 1). The study area encompassed approximately 5900 km^2^ (67°20'–68°20' N, 137°2'–134°50' W), corresponding to the 99% combined kernel home range of all study animals (excluding one dispersing wolf).

The Richardson Mountains are occupied by various other prey species including the migratory Porcupine barren-ground caribou (*Rangifer tarandus granti*), moose (*Alces alces*), muskox (*Ovibos moschatus*), snowshoe hare (*Lepus americanus*), Arctic ground squirrel (*Urocitellus parryii*), and beavers (*Castor canadensis*). Other predators include wolverines (*Gulo gulo*), lynx (*Lynx canadensis*), red foxes (*Vulpes vulpes*), and golden eagles (*Aquila chrysaetos*). The area has no road access. The closest human settlements are Aklavik and Fort McPherson, Northwest Territories.

## Materials and methods

### Animal collaring and monitoring

Collar deployment occurred in autumn 2004 (rams) and spring 2006 (ewes) for Dall sheep, and spring 2006 and 2007 for wolves and grizzly bears. Captures were done from a helicopter by experienced professionals. A handheld net-gun was fired to capture all Dall sheep and most wolves. Dart injection of Telazol, a mixture of tilatemine HCl and zolazapam HCl, was used for some wolves and all grizzly bears. Animals were equipped with GPS collars (Telonics, Mesa, AZ, USA and Lotek Inc., Newmarket, ON, Canada) linked to Argos satellites (CLS America Inc., Largo, MD, USA). Collars deployed in 2004 recorded locations every eight hours. Collars deployed in 2006 and 2007 recorded locations every two hours between May 15 and June 14, to obtain finer resolution of spatial dynamics during lambing season, and every four hours the rest of the year. Each collar had a programmable release mechanism.

All capture, handling, and sampling was approved by the University of Alberta Animal Care and Use Committee for Biosciences in accordance with the Canadian Council on Animal Care guidelines (permit numbers: ACUC412405, ACUC412505, ACUC412605, and ACUC412705). Capture and handling was also approved and conducted under permission from the Government of the Northwest Territories, Department of Environment and Natural Resources, Inuvik Office and the Government of the Northwest Territories Wildlife Care Committee (research permits: WL003119, WL003319, WL005590, and WL007406). Despite use of a net-gun as one of the best options for thinhorn sheep (Kock et al. 1987), one ewe died in a capture accident. All other animal captures were conducted without incident.

Movement data were obtained for 12 Dall sheep (four ewes and eight rams), 15 grizzly bears (10 females and five males), and five wolves (two females and three males). Two additional Dall sheep and four more wolves were collared, but excluded due to collar malfunction (*n* = 3), mortality and harvest (*n* = 2) within weeks of capture. The dispersal movement of one wolf after one year of monitoring was also excluded. During capture sessions and also at periodical collar recovery sessions or field visits, we counted the number of wolves observed together to estimate the number and size of the wolf packs within the study area. Because wolves often hunt in social units [26], we assumed that the space use of the monitored wolves represented that of other pack members.

### Dall sheep seasonal habitat use

Dall sheep seasonal habitat use patterns were estimated from their fixed-kernel utilization distribution constructed with GPS telemetry data [27,28]. To assess temporal variation in resource use and predation risk, we calculated Dall sheep fixed-kernel home ranges for five seasons: winter (November 15 to March 30), spring (April 1 to May 14), lambing (May 15 to June 14), summer (June 15 to August 31), and autumn (September 1 to November 14). For each monitored Dall sheep, habitat use analysis was restricted to its minimum convex polygon home range buffered by one km, to include cells of the utilization distribution located at the edge of the range. This scale reflects choice of habitat features within individual home ranges corresponding to third order selection [29]. We applied standardized methods to select bandwidth (i.e., reference method, least square cross validation [30,31], but results were inconsistent so we manually selected the bandwidth for each species. Dall sheep utilization distributions (bandwidth h = 750) were generated at a 30 m resolution, to match our digital elevation models, using the program Hawth’s Analysis Tools 3.27 (Beyer 2006) for ArcGIS 9.2 (ESRI, Redlands, California, USA). We then employed linear regression models to relate habitat attributes and predation risk, the independent variables, to a probabilistic measure of Dall sheep space use, the dependent variable. This probability was represented by rasterized values of utilization distributions [27] for individual Dall sheep, thereby using each animal as an experimental unit and avoiding pseudoreplication [32]. The method and models have been described at length before [27].

Seasonal utilization distributions for individual Dall sheep were described by topographical features (elevation, slope, aspect, ruggedness), greenness based on the normalized difference vegetation index (NDVI), land cover, and predation risk. Topographical features were calculated from a 30 m grid digital elevation model (Natural Resources Canada). Aspect was categorized as 0 or 1 in the classes N (316°-45°), E (46°-135°), S (136°-225°), W (226°-315°), or nil if the slope was <5°. Terrain ruggedness corresponds to an index of slope and gradient heterogeneity originally developed to study desert bighorn sheep (*O*. *c*. *nelsoni*) habitat selection [33]. The NDVI layer corresponded to mean values for northern Canada from satellite data collected between 1986 and 2006 [34]. We used the 90 m resolution land cover circa-2000 vector [35] and aggregated land cover classes into seven categories (coded as 0 or 1): barrens (moraines, exposed soil, rocks), bryoids (bryophytes and lichens), forests (conifers, deciduous and mixed), herbs (grasses, tussocks, graminoids), shrubs (tall and low), snow or ice, and water (wetlands, lakes and rivers). Land units obscured by clouds or shadows (<2% of the area used by Dall sheep) were omitted from the regression analyses. We interpreted grizzly bear and wolf predation risk as the probability of encountering these species, which was calculated from the predators’ composite utilization distribution (bandwidth value h = 3500 was selected manually, as noted earlier). To assess the influence of predation risk from each species on a seasonal basis, we built season species-specific utilization distributions (five for wolves and four for grizzly bears, which hibernate in winter). Unequal sampling interval within a same season could have resulted in misinterpretation of habitat use patterns. Therefore, to standardize the monitoring frequency of each species throughout the year and yield unbiased utilization distributions [36], we sub-sampled GPS locations at the lowest frequency recorded (eight hours for Dall sheep and four hours for grizzly bears and wolves). Predation risk was estimated as the probability from 0 to 1 that an individual predator is located at a certain point of the Dall sheep seasonal home range at any given time during the season.

Due to collinearity (correlation coefficient > 0.5) with elevation and the barren land cover class, NDVI was excluded from regression models. We present robust estimates of variance (multiplied with N/(N-k), where N is the number of observations and k the number of parameters [37]) and standardized regression coefficients (variance scaled to 1), to compare the relative effect of habitat attributes and predation risk on Dall sheep habitat use, regardless of the variables’ differing units [28]. Regression coefficients (β) for each sex were calculated as the arithmetic mean of individual coefficients for rams and ewes. If a variable coefficient was significantly different from 0 (at α = 0.05), we interpreted that it was either selected for (β > 0) or avoided (β < 0). Rams and ewes exposure to predation risk, based on the coefficient value, were compared using a one-way ANOVA to determine if there was any statistical difference between individuals of each sex. All statistical analyses were conducted in Stata 11.2 (Stata, College Station, Texas, USA).

### Stable isotope analysis

We sampled guard hairs from the forelegs of grizzly bears and wolves handled in 2006 and 2007. For both grizzly bears and wolves, molting occurs annually, starting in late spring to early summer [20,26,38]. Because capture occurred in spring, right before the molt, the collected hair samples should reflect the diet from the previous year [24,38]. For prey, samples were taken from animals handled or carcasses found during fieldwork. Samples were prepared see [39] and the ratios of *δ*^13^C and *δ*^15^N isotopes were analyzed using a continuous-flow ratio mass spectrometry at the Stable Isotopes Facilities (University of Saskatchewan, SK). Results are reported in*δ* notation as deviations in parts per thousands (‰) relative to an international standard (Vienna PeeDee Belemnite (VPDB)).

We analyzed potential food sources known to be used within the study area based on indigenous knowledge and previous studies [40,41]: aquatic browsers (beavers and moose), Arctic ground squirrels, caribou, Dall sheep, fish (Arctic charr (*Salvelinus alpinus*) and broad whitefish (*Coregonus nasus*)), and small rodents (lemmings and microtines). Vegetation was only considered for grizzly bears and included berries, horsetail, grass, sedge, alpine sweetvetch (*Hedysarum alpinum*). Except for Dall sheep samples, which we collected in the field, and Arctic charr values taken from the literature [42], isotopic ratios of sources were taken from locations adjacent to the study area [43]. Since similar isotopic ratios may interfere with our capacity to distinguish between related sources [44], we pooled species to form new groups when similar isotopic signatures existed between prey. None of the monitored individuals traveled as far as the Arctic Ocean during the study so it is unlikely that they relied on additional marine food sources.

Because the isotopic signature of prey tissues is enriched at varying turnover rates within the consumer’s tissues [23,45], we adjusted the *δ*^13^C and *δ*^15^N values of the prey sources to incorporate fractionation rates, also called trophic enrichment [46]. Fractionation is unknown for wolf and grizzly bear hair, so we used rates published for another terrestrial carnivore; the red fox (*Vulpes vulpes*) (-2.6 ± 0.1‰ for *δ*^13^C and 3.4 ± 0.1‰ for *δ*^15^N) [46]. Even if variations between species, tissues, and individuals are well documented [47,48], these rates represent the best available estimates at the time of analysis.

We assessed the contribution of the possible food sources into the predators’ diet using a Bayesian mixing model that allows the inclusion of several sources and their variability, resulting in dietary solutions in terms of true probability distributions [49,50]. The model was built using SIAR (Stable Isotope Analysis in R), based upon a Gaussian likelihood with a mixture Dirichlet-distributed prior on the mean [50,51]. We set uninformative priors for all food sources. For the proportion of each considered food source, SIAR provided a posterior distribution described by a mean and a true probability density function.

## Results

### Dall sheep, grizzly bear and wolf monitoring

Our analyses used 19940 Dall sheep locations (mean ± SE = 1662 ± 328 per individual), 22596 grizzly bear locations (1506 ± 284 per individual), and 11116 wolf locations (2223 ± 364 per individual). We identified three wolf packs that overlapped the Dall sheep range and monitored at least one wolf per pack. Based on ground and aerial observations, pack sizes were 2–8 individuals, although packs were not always cohesive. For stable isotopes analysis, we collected hair samples from 19 grizzly bears, nine wolves, and tissue (hair, heart, kidney and muscle) from 13 Dall sheep.

### Dall sheep seasonal habitat use

**Fig 2:**
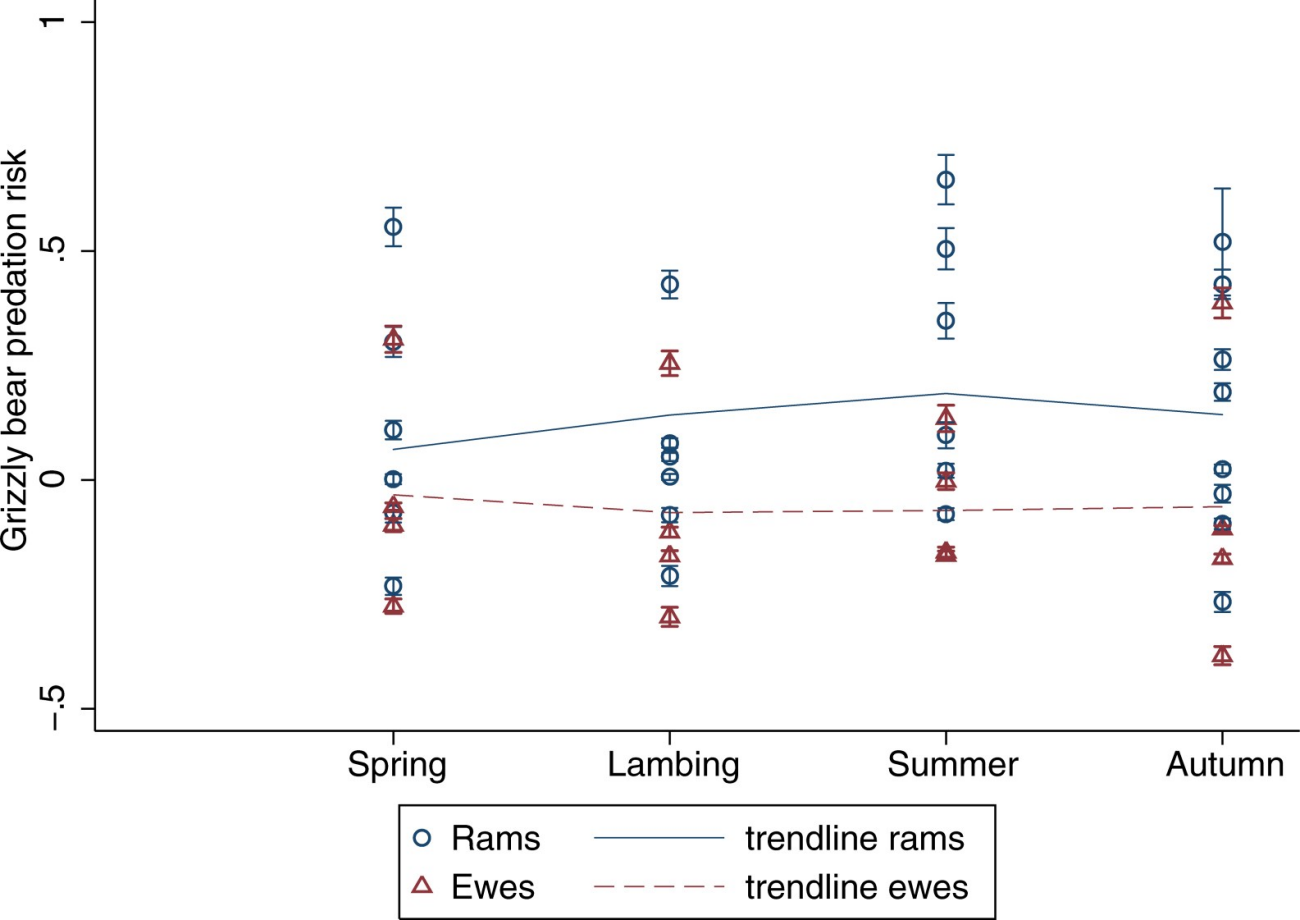
Regression coefficients representing seasonal exposure to grizzly bear predation risk for individual Dall sheep rams and ewes, calculated from the composite seasonal home range of all collared grizzly bears within the study area. Error bars on each data point correspond to the 95% confidence interval. A fractional polynomial trendline for rams and ewes is shown.

**Fig 3:**
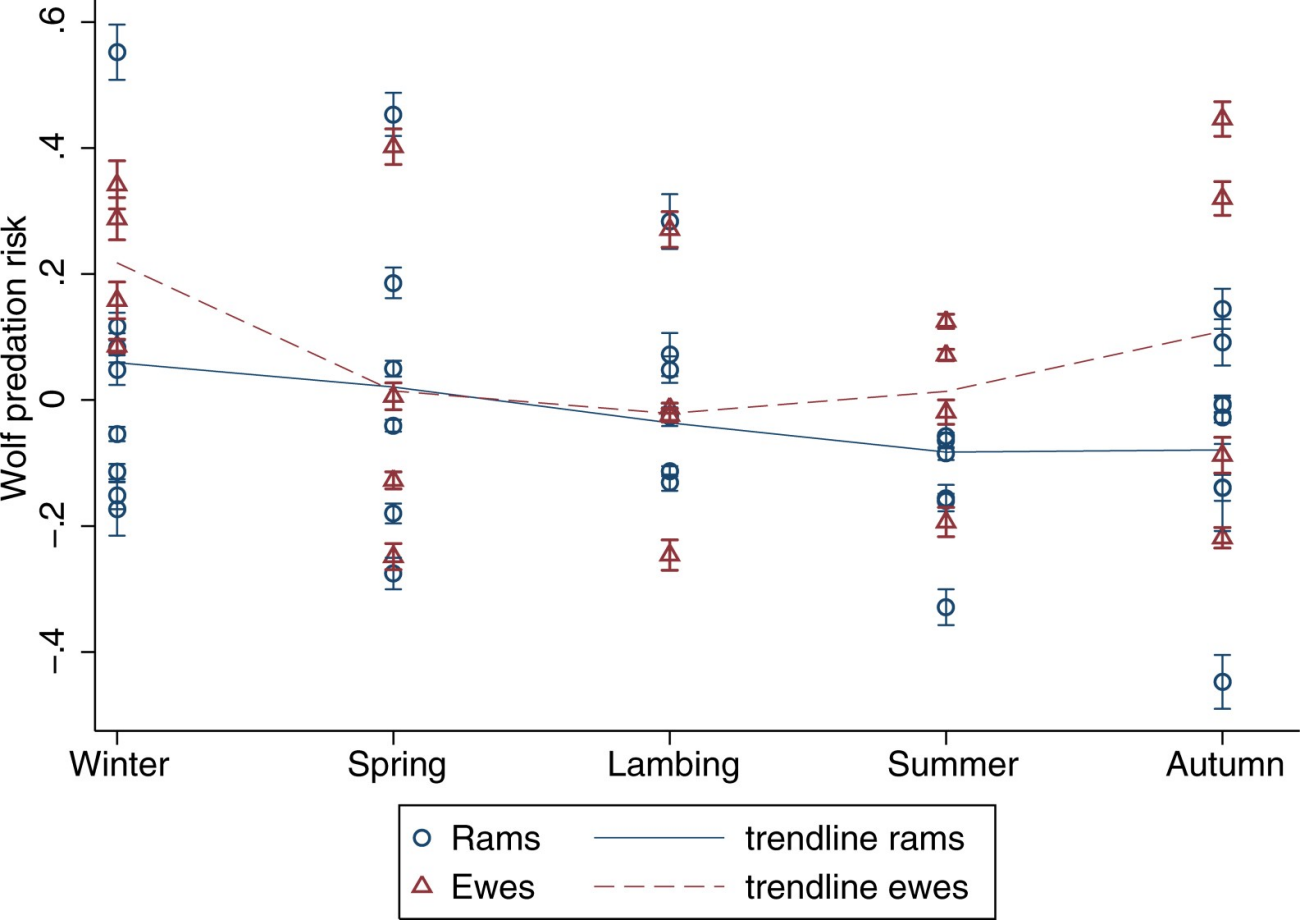
Regression coefficients representing seasonal exposure to wolf predation risk for Dall sheep rams and ewes, calculated from the composite seasonal home range of all collared wolves within the study area. Error bars on each data point correspond to the 95% confidence interval. A fractional polynomial trendline for rams and ewes is shown.

Dall sheep seasonal habitat use varied among individuals but differences between rams and ewes were evident. During winter, rams selected rugged terrain, steep slopes, and barren lands (Table 1). In contrast, ewes selected eastern and western slopes, and were exposed to high wolf risk (Table 2). During spring, rams avoided northern slopes, whilst ewes selected steep slopes. During lambing, rams selected southern slopes as well as land covered by barrens, forests, herbs, shrubs, and water. Ewes selected rugged and steep terrain but avoided northern slopes. During summer, rams avoided northern aspects but selected rugged terrain, steep southeast oriented slopes, as well as barrens, bryoids, and water land covers. Rams were exposed to significantly higher grizzly bear risk, although their exposure to wolves was low (Table 1). Ewes selected higher elevations, rugged and steep terrain, as well as eastern slopes; but avoided south and west oriented aspects, barrens, forests, herbs, shrubs, snow, and water land covers (Table 2). Finally, in autumn, rams selected for habitats at lower elevations, southeast oriented slopes, as well as barrens, bryoids, forests, herbs, shrubs, snow and water land covers (Table 1). No habitat use pattern emerged for ewes in autumn (Table 2). Throughout the year, there was considerable variation in exposure to predation risk among Dall sheep individuals, although rams were overall exposed to higher grizzly bear risk than ewes (*F*_1,40_ = 6.07, *P* = 0.02) (Fig 2). Ewes exposure to wolf risk was highest during winter (Fig 3), but no significant intersexual difference was found (*F*_1,52_ = 2.32, *P* = 0.13).

**Table 1:** Standardized coefficients (β) and standard errors (SE) of seasonal resource utilization for Dall sheep rams. Regression coefficients in bold had confidence intervals that did not include 0, suggesting selection (if β > 0) or avoidance (if β < 0) of specific habitat variables.

Variables	Winter		Spring		Lambing		Summer		Autumn	
	Β	SE	Β	SE	β	SE	β	SE	β	SE
Elevation	-0.039	0.027	0.100	0.064	-0.038	0.073	-0.071	0.048	**-0.211**	0.046
Ruggedness	**0.063**	0.032	0.081	0.054	0.028	0.028	**0.082**	0.018	0.029	0.030
Slope	**0.048**	0.024	0.036	0.042	0.008	0.026	**0.081**	0.033	0.011	0.029
N	-0.012	0.045	**-0.102**	0.038	-0.056	0.038	**-0.063**	0.025	0.006	0.026
E	-0.010	0.041	-0.029	0.041	-0.021	0.037	**0.031**	0.005	**0.049**	0.021
S	0.014	0.022	0.034	0.025	**0.042**	0.016	**0.041**	0.011	**0.098**	0.032
W	-0.009	0.021	-0.018	0.014	-0.022	0.014	0.025	0.022	0.019	0.014
Barrens	**0.143**	0.069	-0.082	0.084	**0.202**	0.079	**0.184**	0.064	**0.463**	0.073
Bryoids	0.019	0.029	0.000	0.058	0.035	0.034	**0.088**	0.042	**0.122**	0.040
Forests	0.059	0.037	-0.098	0.053	**0.127**	0.056	0.021	0.042	**0.201**	0.056
Herbs	0.114	0.079	-0.125	0.094	**0.240**	0.090	0.119	0.081	**0.455**	0.091
Shrubs	0.074	0.053	-0.101	0.070	**0.172**	0.061	0.093	0.052	**0.299**	0.065
Snow	0.042	0.038	-0.046	0.058	0.040	0.044	-0.028	0.059	**0.103**	0.035
Water	0.026	0.016	-0.009	0.014	**0.017**	0.008	**0.061**	0.017	**0.065**	0.012
Wolf risk	0.038	0.083	0.032	0.107	0.022	0.062	**-0.142**	0.042	-0.066	0.065
Grizzly risk	NA	NA	0.110	0.115	0.046	0.087	**0.258**	0.118	0.129	0.095

**Table 2:** Standardized coefficients (β) and standard error (SE) of seasonal resource utilization for Dall sheep ewes. Regression coefficients in bold had confidence intervals that did not include 0, suggesting selection (if β > 0) or avoidance (if β < 0) of specific habitat variables.

Variables	Winter		Spring		Lambing		Summer		Autumn	
	Β	SE	Β	SE	β	SE	β	SE	β	SE
Elevation	-0.075	0.052	-0.032	0.080	0.021	0.048	**0.286**	0.035	0.038	0.179
Ruggedness	-0.020	0.023	0.062	0.055	**0.184**	0.055	**0.090**	0.031	0.012	0.046
Slope	0.020	0.078	**0.054**	0.016	**0.127**	0.032	**0.121**	0.044	0.003	0.033
N	0.075	0.040	-0.041	0.031	**-0.063**	0.032	-0.062	0.032	-0.021	0.025
E	**0.127**	0.051	0.075	0.087	0.006	0.031	**0.085**	0.013	0.060	0.037
S	0.037	0.041	0.018	0.017	0.037	0.049	**-0.022**	0.010	0.030	0.039
W	**0.055**	0.012	-0.041	0.022	0.010	0.026	**-0.073**	0.017	-0.024	0.030
Barrens	0.119	0.116	0.190	0.173	0.060	0.086	**-0.395**	0.109	0.234	0.369
Bryoids	0.001	0.030	0.022	0.029	-0.038	0.053	-0.125	0.067	-0.050	0.049
Forests	0.003	0.036	0.003	0.071	-0.071	0.060	**-0.214**	0.077	-0.052	0.094
Herbs	0.076	0.113	0.143	0.165	-0.004	0.080	**-0.484**	0.092	0.184	0.356
Shrubs	0.027	0.068	0.075	0.080	-0.017	0.040	**-0.256**	0.049	0.052	0.196
Snow	0.011	0.035	0.028	0.036	-0.017	0.038	**-0.082**	0.038	-0.035	0.057
Water	0.036	0.025	0.023	0.020	0.010	0.008	**-0.062**	0.007	0.016	0.045
Wolf risk	**0.218**	0.059	0.008	0.141	-0.003	0.106	-0.004	0.070	0.115	0.159
Grizzly risk	NA	NA	-0.031	0.122	-0.081	0.119	-0.048	0.071	-0.069	0.163

### Dall sheep in grizzly bear and wolf diets

**Fig 4:**
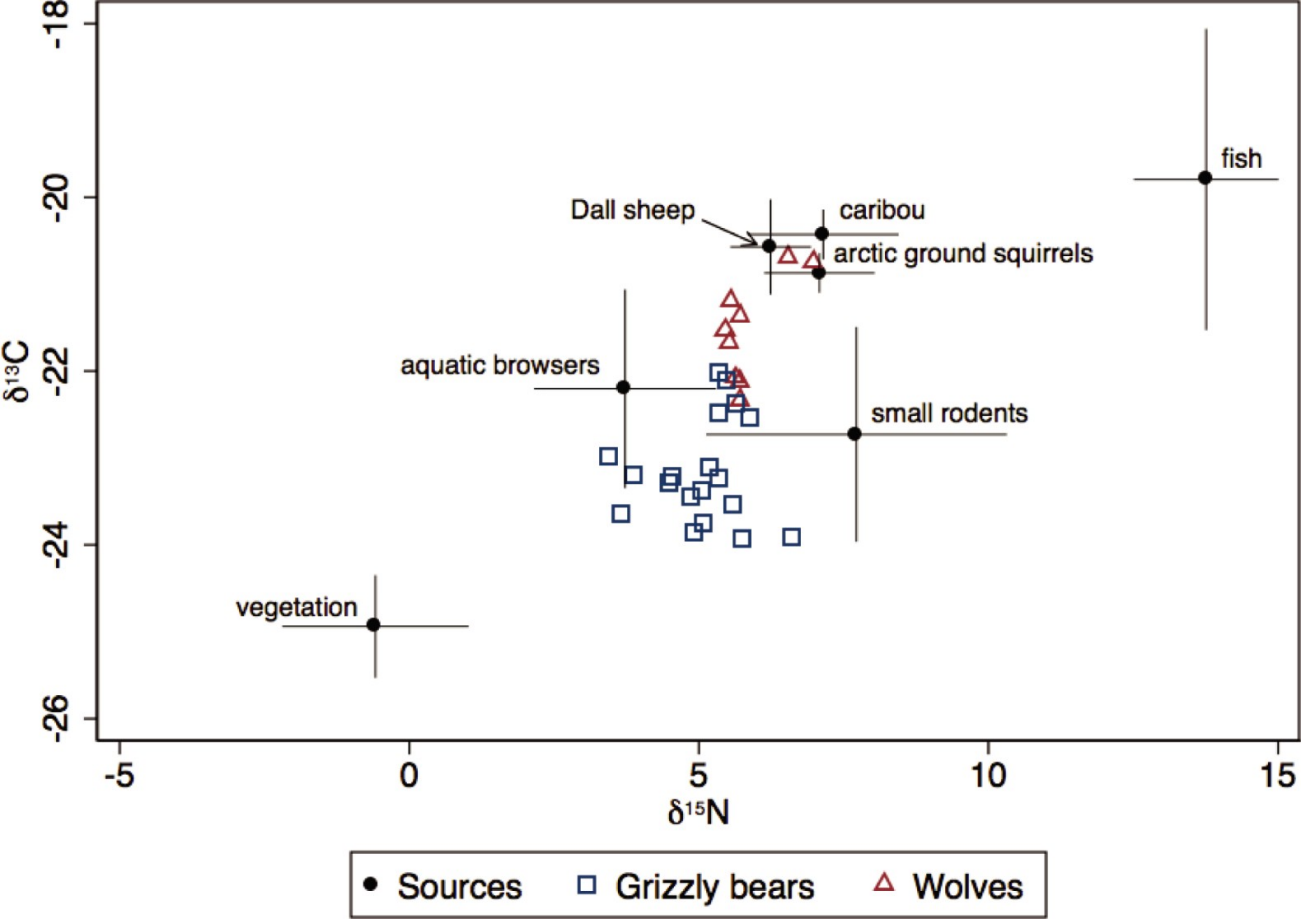
*δ*^13^C (‰) and *δ*^15^N (‰) stable isotope signatures for grizzly bears and wolves. Food sources values were adjusted to account for fractionation, and cross bars on food sources show standard deviations.

**Fig 5:**
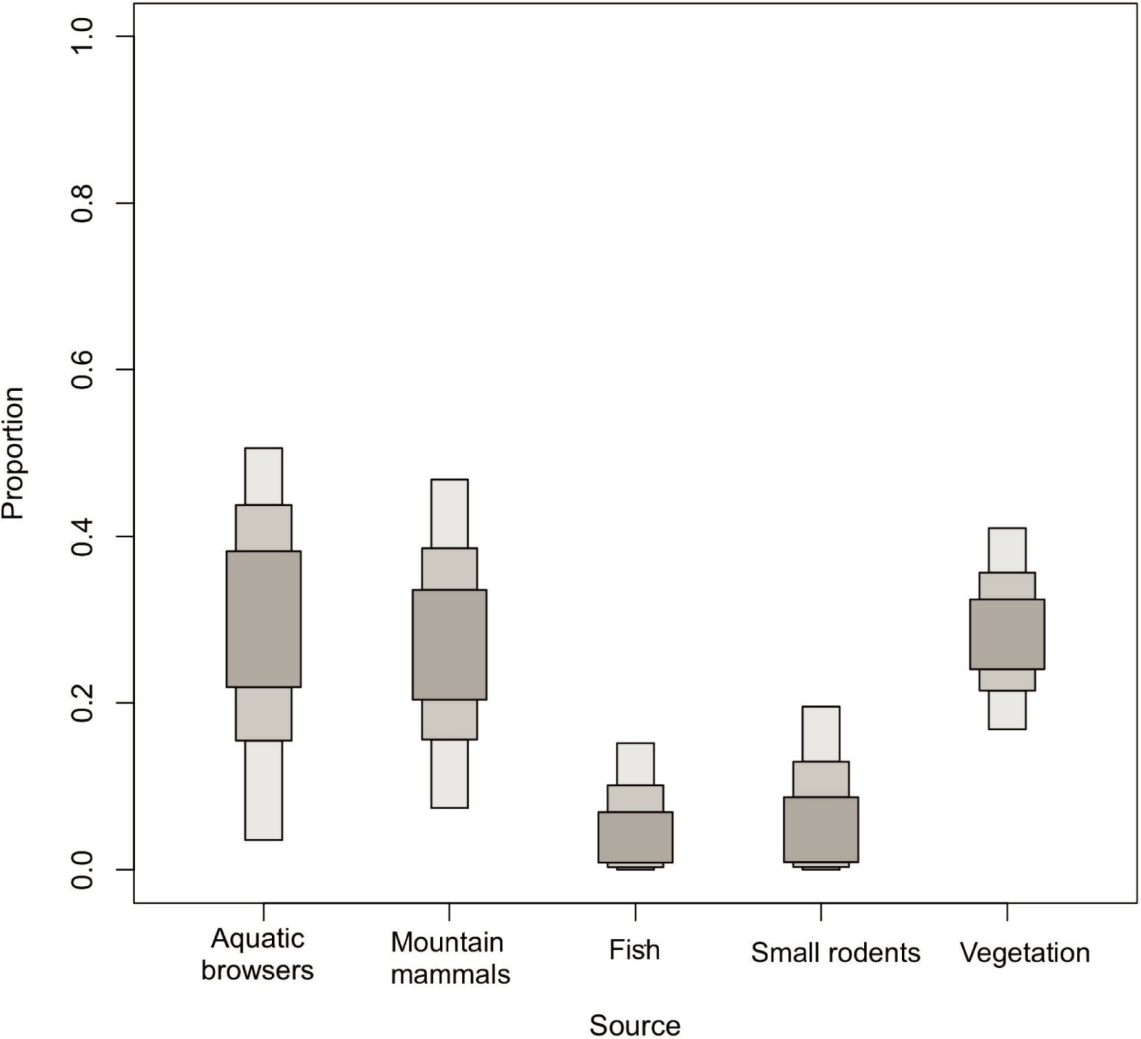
Estimated proportions of various food source groups in the diet of grizzly bears, after combining Dall sheep, caribou, and Arctic ground squirrels in the mountain mammals group (*a posteriori* model). Darker, medium and lighter grey bars respectively indicate the 25, 75, and 95% credible intervals.

**Fig 6:**
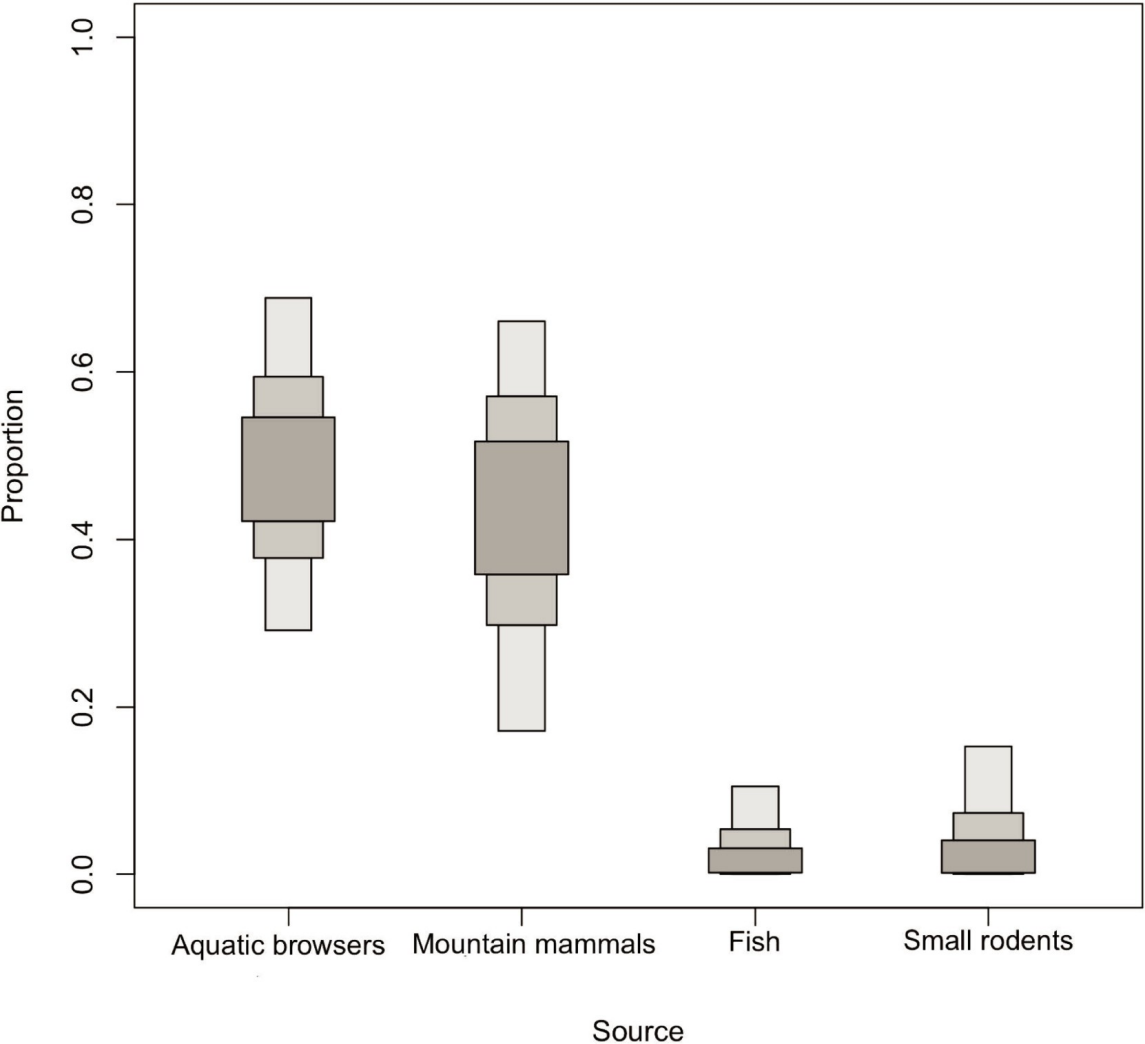
Estimated proportions of various food source groups in the diet of wolves, after combining Dall sheep, caribou, and Arctic ground squirrels in the mountain mammals group. Darker, medium and lighter grey bars respectively indicate the 25, 75, and 95% credible intervals.

Stable isotope values for grizzly bears and wolves reflected a range of signatures. Mean carbon and nitrogen isotope values (± SE) were respectively -23.17 ± 0.14‰ *δ*^13^C (range -23.93 to -22.02) and 5.07 ± 0.18‰ *δ*^15^N (range 3.46 to 6.61) for grizzly bears, and -21.53 ± 0.20‰ *δ*^13^C (range -22.34 to -20.69) and 5.87 ± 0.17‰ *δ*^15^N (range 5.46 to 6.98) for wolves (Fig 4). Because Dall sheep isotopic signature was similar to that of caribou and Arctic ground squirrels (Fig 4), there was no clear way to differentiate between them and so we pooled these three species into a group called *mountain mammals*. This model contained five groups of food sources and yielded a mean dietary proportion of 27.5% mountain mammals for grizzly bears (Fig 5), and 42.5% for wolves (Table 3, Fig 6).

**Table 3:** Mean proportion and 95% credible interval (CI) of various food sources in the assimilated diet of grizzly bears and wolves, after Dall sheep, Arctic ground squirrels, and caribou were merged into mountain mammals.

Source	Grizzly bears		Wolves	
	Mean (%)	95% CI	Mean (%)	95% CI
Aquatic browsers	28.6	3.8–50.0	48.3	29.0–69.0
Mountain mammals	27.5	8.3–47.0	42.5	17.0–66.0
Fish	6.7	0.0–15.0	3.8	0.0–10.0
Small rodents	8.6	0.0–20.0	5.3	0.0–15.0
Vegetation	28.6	17.0–41.0	NA	NA

The most important food source groups for grizzly bears were vegetation, aquatic browsers (beavers and moose), and mountain mammals. On average, animal sources accounted for approximately 70% of the grizzly bear diet (Table 3). For wolves, their principal food source was aquatic browsers and mountain mammals. Fish and small rodents were the least used for both species (Table 3).

## Discussion

Sexual segregation in this Dall sheep population was evident in the different seasonal habitat use patterns of rams and ewes. Throughout the year, habitat features most often selected by rams were barrens, followed by water and southerly aspects, then by ruggedness, slope, avoidance of northerly aspects, use of bryoids, forests, herbs, and shrubs. All of these categories, except ruggedness and slope, are likely linked to ground vegetation and foraging. Dall sheep are primarily grazers of grasses and sedges, but also browses, forbs, moss, and lichens [52,53]. It is likely that the barrens, which rams used in four out of five seasons, provided sparse vegetation and wind-swept ridges to access to forage during winter or slopes that are critical to insect avoidance during harassment periods in northern ungulates [54]. Associations with water features from lambing to autumn may be linked to the dryness of this environment after the snowmelt. Finally, the use of south-facing and avoidance of north-facing slopes are likely related to the availability of better forage on slopes with maximal sun exposure as well as the need to minimize body heat loss in this northern ecosystem [6]. For rams, predation risk from grizzly bears was higher in the summer, which coincided with lower wolf predation risk.

In contrast, habitat variables most often selected by ewes were steep slopes, followed by ruggedness and eastern aspects. The use of steep or rugged escape terrain is a well-documented predator avoidance behaviour of Dall sheep [5,7,55,56]. Ewes selected steep slopes and rugged terrain during lambing and summer, when lambs are most vulnerable to predation. In the northern Richardson Mountains, the importance of safety for ewes was reflected in their habitat use, at the cost of land cover that could provide higher foraging. Dall sheep ewes were overall less exposed than rams to grizzly bear predation, although their exposure to wolf predation increased during winter–when lambs are less vulnerable and factors like foraging and thermoregulation may gain importance.

Dall sheep seasonal habitat use analyses revealed patterns that varied for rams and ewes. Foraging needs emerged as a key factor affecting the rams’ habitat choices, whereas ewes’ habitat choices seemed to be motivated by predator avoidance–particularly when lambs are young and most vulnerable. Our results suggest that predation risk plays an important role in Dall sheep habitat use and sexual segregation. Our study also demonstrates that Dall sheep individuals were exposed to various levels of risk throughout the year. Specifically, ewe exposure to wolf predation risk peaked during the winter; whereas rams were more at risk to grizzly bear predation than ewes, with an increased exposure during the summer.

The wide confidence intervals of isotopic signatures indicated individual variation in dietary habits of grizzly bears in the Richardson Mountains, although to a lesser extent than in the adjacent Mackenzie Delta, where distinct foraging profiles were identified [24]. Over two-thirds of the grizzly bears’ assimilated diet were from animal sources dominated by aquatic browsers (beavers and moose). Grizzly bear predation on moose has been reported in few populations [57,58,59]. Dall sheep, caribou, and Arctic ground squirrels composed altogether over one-quarter of their diet. During the study, bears were observed chasing Dall sheep as well as feeding on their carcasses, although the Dall sheep proportion in their diet cannot be distinguished from other mountain mammals with stable isotopes. For several coastal bear populations, fish is a major food source [23,60]. In the Mackenzie Delta, grizzly bears feed on broad whitefish [61] but in the Richardson Mountains, our results suggest that the fish consumption was minimal.

Given the individual variation in isotopic signatures among this population, it is likely that some wolves rely more on Dall sheep than others. Variation in the diet of wolves can be a consequence of some individuals specializing on certain prey [21,62]. Specialized predators, however, can lead to stochastic predation events that may adversely affect mountain sheep populations [63]. The wolves’ main food sources in the Richardson Mountains appeared to be aquatic browsers followed closely by mountain mammals. Wolves in central Yukon relied primarily on moose [64], wolves in northwest Alaska preyed almost equally on moose and caribou [65], and wolves in Nunavut mostly followed migratory barren-ground caribou [66]. In the Richardson Mountains, wolves prey on the Porcupine caribou herd, although kill rates greatly varied among packs [67]. In our study, caribou were part of the wolves’ diet (based on carcasses and field observations) but we did not observe any migratory movements of wolves into the calving grounds, which are located on the coastal plain [68]. Our collar data suggest that wolves of the Richardson Mountains are resident because they mostly stayed all year within their home range.

Our results suggest that protein is critical to this grizzly bear population and that mountain mammals, likely including Dall sheep, constitute an important part of both grizzly bears’ and wolves’ diets. Due to their close isotopic signatures, methods other than carbon and nitrogen stable isotopes analysis are required to distinguish the exact dietary proportion of Dall sheep from caribou and Arctic ground squirrels, particularly given their spatial proximity. Holders of Gwich’in and Inuvialuit ecological knowledge have also mentioned that vegetation (mostly berries) and Arctic ground squirrels are important dietary components for grizzly bears near Dall sheep groups [69,70]. Overall, wolves and grizzly bears both appear to be consuming Dall sheep, although not as their main prey species and with variation between individuals. To determine the contribution of grizzly bears and wolves to Dall sheep abundance fluctuations, direct monitoring of predation events and mortality rates would be needed. Likewise, the isotopic analyses alone cannot tell if Dall sheep were eaten as a fresh catch or as a carrion. In addition to predation, factors like human harvest, climate, and density-dependence all likely play a role in limiting this population. Although very few diseases have been reported until now, protostrongylid parasites and pneumonia outbreaks may become a threat with global warming and the encroachment of domestic sheep near wild thinhorn sheep herds [71].

Predator-prey interactions are intricate and challenging to investigate, particularly in remote ecosystems and for species with large home ranges. This paper contributed to our understanding of Dall sheep, grizzly bear, and wolf interactions in the northern Richardson Mountains through a combination of approaches. Our results revealed important patterns related to Dall sheep seasonal habitat utilization, sexual segregation, predation risk, and delineated grizzly bear and wolf diets. This study confirms the importance of considering apparent predation risk when investigating prey habitat use patterns. Using such combination of spatial analyses and stable isotopes analyses could be beneficial in a wide range of ecosystems to better assess the potential role of predation on prey.
